# Effects of cannabidiol on behavioral and psychological symptoms of vascular dementia: a randomized, double-blind, placebo-controlled trial.

**DOI:** 10.1192/j.eurpsy.2023.250

**Published:** 2023-07-19

**Authors:** R. M. D. P. P. Pessoa, M. H. N. Chagas

**Affiliations:** 1 University of São Paulo, Ribeirão Preto; 2 Bairral Institute of Psychiatry, Itapira, Brazil

## Abstract

**Introduction:**

Vascular dementia (VD) is the second most common cause of dementia and is characterized by cerebrovascular changes causing cognitive impairment. In patients with VD, behavioral and psychological symptoms of dementia (BPSD) are heterogeneous and highly prevalent manifestations that can arise in the course of dementia and bring suffering to the individual and his family. Currently, pharmacological interventions in the treatment of these symptoms have important adverse effects. Cannabidiol (CBD) has neuroprotective, anxiolytic and antipsychotic properties, as well as a favorable tolerability and safety profile.

**Objectives:**

To evaluate the effect of CBD on behavioral and psychological symptoms in elderly with VD.

**Methods:**

Double-blind, randomized, placebo-controlled clinical trial involving elderly patients with VD. The instruments used are: Neuropsychiatric Inventory, Brief Psychiatric Rating Scale (BPRS), Clinical Global Impression Scale, Side Effects Scale, Mini- Mental State Examination, Brief Cognitive Screening Battery, Katz Index of Independence in Activities of Daily Living, Lawton Instrumental Activities of Daily Living Scale, Informant Questionnaire on Cognitive Decline in the Elderly, Zarit Burden Inventory. Included participants were assessed at baseline, at the first, second, and fourth weeks after the start of the clinical trial.

**Results: Parcial results:**

Up to the present moment, 18 patients were included, eight in group 1 and ten in group 2. In the initial evaluation of the BPSD, the mean in the Neuropsychiatric Inventory was 43.25 (±21.89) in group 1 and 50 (±18.86) in group 2, and in the BPRS, the mean was 25.25 ( ±9.82) in group 1 and 34.30 (±15.11) in group 2. The final BPRS averaged 23 (±11.41) in group 1 and 20.40 (±13.32) in group 2. The Neuropsychiatric Inventory averaged in the final assessment 41.88 (±20.15) and 17.60 (±12.33) in groups 1 and 2, respectively.

**Conclusions:**

Cannabidiol has been shown to be a strategy for the management of BPSD. In addition, it has a good side-effect profile.

**Disclosure of Interest:**

None Declared

